# The influence of fake news on face-trait learning

**DOI:** 10.1371/journal.pone.0278671

**Published:** 2022-12-21

**Authors:** Adam Eggleston, Richard Cook, Harriet Over

**Affiliations:** 1 Department of Psychology, University of York, York, United Kingdom; 2 The School of Psychology, University of Leeds, Leeds, United Kingdom; Roma Tre University: Universita degli Studi Roma Tre, ITALY

## Abstract

Humans spontaneously attribute a wide range of traits to conspecifics based on their facial appearance. Unsurprisingly, previous findings indicate that this ‘person evaluation’ is affected by information provided about the target’s past actions and behaviours. Strikingly, many news items shared on social media sites (e.g., Twitter) describe the actions of individuals who are often shown in accompanying images. This kind of material closely resembles that encountered by participants in previous studies of face-trait learning. We therefore sought to determine whether Twitter posts that pair facial images with favourable and unfavourable biographical information also modulate subsequent trait evaluation of the people depicted. We also assessed whether the effects of this information-valence manipulation were attenuated by the presence of the “disputed tag”, introduced by Twitter as a means to combat the influence of fake-news. Across two preregistered experiments, we found that fictional tweets that paired facial images with details of the person’s positive or negative actions affected the extent to which readers subsequently judged the faces depicted to be trustworthy. When the rating phase followed immediately after the study phase, the presence of the disputed tag attenuated the effect of the behavioural information (Experiment 1: N = 128; *M*_age_ = 34.06; 89 female, 36 male, 3 non-binary; 116 White British). However, when the rating phase was conducted after a 10-minute delay, the presence of the disputed tag had no significant effect (Experiment 2: N = 128; *M*_age_ = 29.12; 78 female, 44 male, 4 non-binary, 2 prefer not to say; 110 White British). Our findings suggest that disputed tags may have relatively little impact on the long-term face-trait learning that occurs via social media. As such, fake news stories may have considerable potential to shape users’ person evaluation.

## Introduction

Adults spontaneously attribute a wide range of traits to others based on their facial appearance. For example, making judgments about their apparent trustworthiness, honesty, competence, intelligence, and likeability [1, 2]. These first impressions are thought to load on at least two principal dimensions–often referred to as trustworthiness or valence and dominance [1, 3] and are formed quickly, sometimes within 100 milliseconds of meeting another person [4–6]. Although individuals within a culture often form similar first impressions, judgments from appearance cues alone have little or no basis in reality, rarely reflecting the actual traits of the individuals being judged [7–10]. Nevertheless, these judgements exert a powerful influence over behaviour and decisions: Individuals judged competent are more likely to be elected to public office [2, 4], while individuals judged trustworthy are more likely to receive lenient sentences in criminal justice situations [11].

Unsurprisingly, this ‘person evaluation’ is influenced by information about the target’s past actions and behaviours [12–15]. Indeed, even a single behavioural statement is sufficient to influence perceptions of another person’s trustworthiness. For example, faces that have been paired with a positive behaviour (e.g., “Gave his balloon to a child who had let hers go”) are judged more trustworthy than those that have been paired with a negative behaviour (e.g., “Stole money and jewellery from the relatives he was living with”) [13]. Interestingly, in line with the Associative-propositional evaluation model, learning of this sort generalises to novel targets with a similar appearance [16–20]. Attempts to modify person perception have yielded mixed results. Learned associations between appearance and character can be difficult to override as new experiences do not necessarily cancel out old associations [21–25]. Due to the ease with which face-trait learning takes place, the potential for it to transfer to novel targets, and its seeming resistance to counter training, it is crucial to understand the real-world implications of face-trait learning.

Social media sites may be one source of face-trait learning. Sites such as Twitter are becoming increasingly popular sources of news and current affairs [26, 27]. The success of these platforms is in part attributable to the ease with which users can share news items that affect themselves, their friends, and family. Depending on the platform and the size of the user’s network, shared items can be viewed by thousands of others in a matter of minutes. Many of the news items shared on social media sites describe the actions and behaviours of individuals. Frequently, these news stories are accompanied by a picture of the person’s face. This format closely approximates that employed in lab-based studies of face-trait learning [12–15]. Given the results of these studies, it is likely that social media posts pairing facial images and behavioural information will affect the evaluation of the individuals depicted by users who encounter this content.

Given the reach of social media and the speed with which items can be shared across a network, posts that pair faces and trait relevant biographical information potentially exert a powerful influence on the public’s perception of the individuals depicted. Alarmingly, however, much of the information shared online is misleading or even inaccurate [28]. Indeed, fake news appears to spread faster and further online than stories verified to be true [29]. There is a clear danger, therefore, that people will be unfairly evaluated because of misleading information shared on social media. Moreover, this possibility could be exploited as an instrument of propaganda, used to tarnish the perception of a political rival or improve perception of a favoured candidate [30].

The proliferation of fake news is a concern for several reasons. For example, fake news is thought to hinder the success of public health programmes [31, 32] and undermine democratic elections [33–35]. In response, social media platforms are attempting to mitigate the effects of fake news via several means including the prioritisation of news from sources judged trustworthy [36], the incorporation of web plug-ins that quickly highlight sites known to spread fake news [37], and the removal of unreliable accounts [36, 38]. Similarly–and of particular relevance to the present investigation–sites including Twitter have started adding a “disputed tag” to flag news stories that may be inaccurate or unreliable.

Several studies have shown that participants are able to rationally incorporate information about the credibility of sources when deciding what to believe [39, 40]. For example, the presence of a disputed tag significantly decreases participants’ perception of an article’s believability and accuracy [41–43] even up to a week later [44]. However, recent research suggests that repeated exposure to a fake news story increases its believability even when a disputed tag is present [45, 46]. Furthermore, work by Baum and colleagues [14, 47] suggests that trait-relevant information influences person evaluation irrespective of source credibility. For example, when asked to evaluate faces that had been paired with favourable or unfavourable news headlines, participants’ judgements were based on the valence of the headline even when the source was distrusted [14]. Similalry, faces paired with negative biographical details (e.g., “he bullied his apprentice”) were judged untrustworthy even when the information was qualified by the addition of “allegedly” [47].

The present investigation had two aims: First, we sought to determine whether Twitter posts that pair facial images with favourable and unfavourable biographical information modulate person evaluation in a manner consistent with previous empirical findings [12–15]. In line with these previous findings, we predicted that targets displayed with a negative headline would subsequently be judged as less trustworthy than targets displayed with a positive headline. Our second aim was to establish whether the effects of the information-valence manipulation were attenuated by the presence of the “disputed tag”. Some previous research has shown that participants are able to utilise information on source credibility in their decision making [39, 40], given the ease with which face-trait learning occurs [4] and previous work on the robustness of this learning [14, 47], it is also possible that a target’s trustworthiness will be influenced by a tweet’s valence even when the information within it is marked as disputed.

## Experiment 1

In Experiment 1, we presented participants with displays pairing target faces with positively or negatively valanced headlines that either appeared with or without a disputed tag. During this study phase, we asked participants how believable they found each headline. This served as a manipulation check to ensure that participants were attending to and encoding the disputed tag. In the subsequent test phase, we presented the target faces in isolation (i.e., in the absence of the tweet context and the news headline) and asked participants to judge their perceived trustworthiness. After the crucial test phase, we also measured whether participants could recall whether the headline associated with each face was positive or negative, disputed or non-disputed.

### Method

#### Participants

Protocols were approved by the University of York’s Psychology Ethics Committee, participants gave informed written consent before taking part. One-hundred-and-twenty-eight participants completed the experiment based on a power analysis using MorePower 6.0.4 that found a minimum *N* of 126 would be necessary to detect interactions with a medium effect size (partial eta squared .06) with an alpha of .05 and power of .8 (*M*_age_ = 34.06, *SD*_age_ = 12.93; 89 female, 36 male, 3 non-binary). All participants were recruited via the online platform Prolific (www.prolific.co). A further 42 were tested, but replaced having reported that they did not notice the disputed tag. Of the 128 participants in the final sample, all reported English as their first language and 126 resided in the UK (2 selected ‘prefer not to say’). Of these 128 participants, 116 identified as White British, 2 as “Other” White background (not-specified), 4 as Indian, 2 as Chinese, 1 as Irish, 1 as Caribbean, and 1 as Pakistani. All participants received a small honorarium (£2.50) for their participation.

#### Materials

In a pre-test, we asked 30 participants, recruited via Prolific, to rate 60 headlines (30 positive, 30 negative) on positivity and on 3 items related to credibility (authenticity, believability, accuracy). All scales ran from -50 to +50. Based on these scores, the 12 most credible headlines of each valence were chosen for use in the current experiment. All positive headlines had a positivity rating above +25, while all negative headlines had a positivity rating of less than -25.

The target stimuli used in this experiment were photographs of AI generated faces taken from the openly available Academic Dataset by Generated Photos (https://generated.photos/datasets). This database was chosen as it provided a large number of high quality and constrained images of forward-facing faces featuring a range of ethnicities, genders and ages. A subset of 100 (50 male, 50 female) adult faces were chosen from the database based on their neutral expression and forward-facing head position.

In a separate pre-test, we recruited two groups of 60 participants to rate the 100 faces on measures of trustworthiness and attractiveness (both scales: -50 to +50) as well as age (scale: under-18 to over-60). Thirty participants rated the male faces, 30 participants rated the female faces. Based on the mean ratings for each measure we created four sets of 6 faces (3 male, 3 female) with each set closely matched on all three measures.

**Fig 1:**
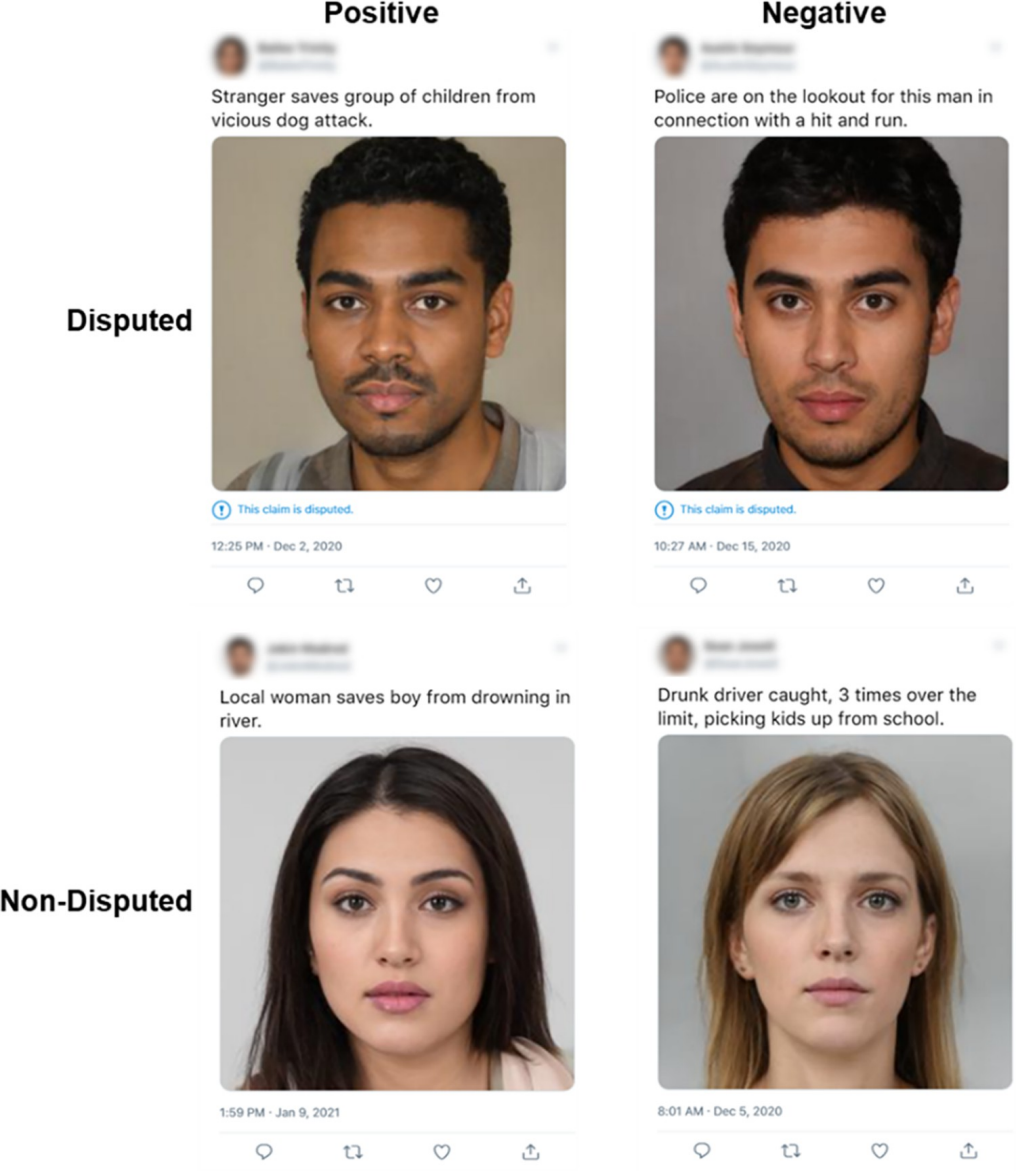
Example tweet stimuli. Stimuli encountered by participants during the study phase. Face stimuli taken from Generate Photos (https://generated.photos/datasets) and tweets created in (www.tweetgen.com).

Combinations of facial images and headlines were transformed into tweets using an online fake tweet generator (www.tweetgen.com) with or without a disputed tag (Fig 1). A full list of the headlines, stimuli and all ratings can be found in “Supplementary materials” at the Open Science Framework: (https://osf.io/t7c25/?view_only=8e6ca4f1249c4579a0e49e1ce5f4d747).

#### Design and counterbalancing

During the study phase, participants observed fictious news tweets that were positive and negative disputed and not disputed in a 2 × 2 factorial design. In total, participants viewed 24 tweets, six for each of the four factorial combinations. Each tweet was presented twice across two blocks, once in block-1 and once in block-2, yielding 48 study trials. The order in which all tweets were presented within each block was randomised. The allocation of face set (1, 2, 3, or 4) to factorial combination (positive disputed, positive non-disputed, negative disputed, negative non-disputed) was perfectly counterbalanced across the sample.

During the test phase, all participants completed the same rating procedure, in which the 24 faces were presented in a randomised order. Each face was rated once, yielding 24 test trials. In the subsequent recall phase, the order of two final questions used to assess participants’ memory for headline valence and disputed status was counterbalanced.

#### Procedure

At the start of the study phase, participants were informed that they would be shown “a series of tweeted news stories” and that “each tweet contains a picture of the person referred to in the headline.” They were asked to rate how believable they considered the tweet to be using a sliding scale that appeared below the statement: “I think the tweet I just read is believable”. The scale ranged from -50 ("Describes the tweet very poorly") to +50 ("Describes the tweet very well"). Participants were unable to see the numerical value they selected on the slider. Each tweet was visible for 3 secs before the question and rating scale appeared. The tweet remained on screen until participants had entered their rating and clicked to proceed to the next trial.

During the test phase, target faces (without headlines or tweet context) appeared one at a time in the centre of the screen. Underneath the facial image was a question prompt: “How trustworthy do you think this person is?” Participants recorded their judgements using a scale that ranged from -50 (“Not at all Trustworthy”) to +50 (“Extremely Trustworthy”). Participants were unable to see the numerical value they selected on the slider. Faces were visible until a response was recorded.

In the penultimate recall phase, participants again viewed each of the target faces, one at a time. For each face, they were asked two questions in a counterbalanced order: The first was whether or not the face was paired with a positive or a negative headline. Participants responded to the statement “The headline paired with this face was positive” using a scale ranging from -50 ("Not at all Confident") to +50 ("Extremely Confident"). The second was whether or not the associated headline had a disputed tag present. Participants responded to the statement “The headline paired with this face was disputed” using a scale ranging from -50 ("Not at all Confident") to +50 ("Extremely Confident").

Finally, participants were asked to complete a short demographic questionnaire recording gender, ethnicity, age, twitter use and whether or not they had noticed that some of the tweets contained a disputed tag. After participants had completed all parts of the questionnaire they were thanked and debriefed. The experiment was conducted using Gorilla Experiment Builder (https://gorilla.sc). Participants’ reported twitter use and examples of all instruction and response screens can be found in Supplementary materials at the Open Science Framework (https://osf.io/t7c25/?view_only=8e6ca4f1249c4579a0e49e1ce5f4d747).

### Results

The data for this experiment, as well as our pre-registered hypotheses and analysis plans, are available open access at Open Science Framework (https://osf.io/t7c25/?view_only=8e6ca4f1249c4579a0e49e1ce5f4d747).

#### Believability ratings

The believability ratings obtained during the study phase were subjected to ANOVA with Valence (positive, negative), Credibility (disputed, non-disputed) and Presentation (first, second) as within-subjects factors. As expected, there was a significant main effect of Credibility [*F*(1,127) = 130.80, *p* < .001, η_p_^2^ = .34], whereby non-disputed tweets (*M* = 18.37) were rated as more believable than disputed tweets (*M* = -7.57). There was also a significant main effect of Valence [*F*(1,127) = 103.63, *p* < .001, η_p_^2^ = .10], whereby positive tweets (*M* = 12.30) were rated as more believable than negative tweets (*M* = -1.51). The analysis revealed a significant Credibility × Presentation interaction [*F*(1,127) = 11.90, *p* < .001, η_p_^2^ = .002]. However, the believability of disputed tweets at presentation one (*M* = -6.49) and presentation two (*M* = -8.66) did not differ significantly (*p* = .158). Similarly, the believability of non-disputed tweets at presentation one (*M* = 17.33) and presentation two (*M* = 19.41) did not differ significantly (*p* = .200). There was no main effect of Presentation [*F*(1,127) = .004, *p* = .951, η_p_^2^ = < .001], no Valence × Credibility interaction [*F*(1,127) = 1.55, *p* = .216, η_p_^2^ =.xx], no Valence × Presentation interaction [*F*(1,127) = 1.16, *p* = .284, η_p_^2^ = < .001], and no Valence × Credibility × Presentation interaction [*F*(1,127) = 1.08, *p* = .300, η_p_^2^ = < .001].

#### Trustworthiness ratings

**Fig 2:**
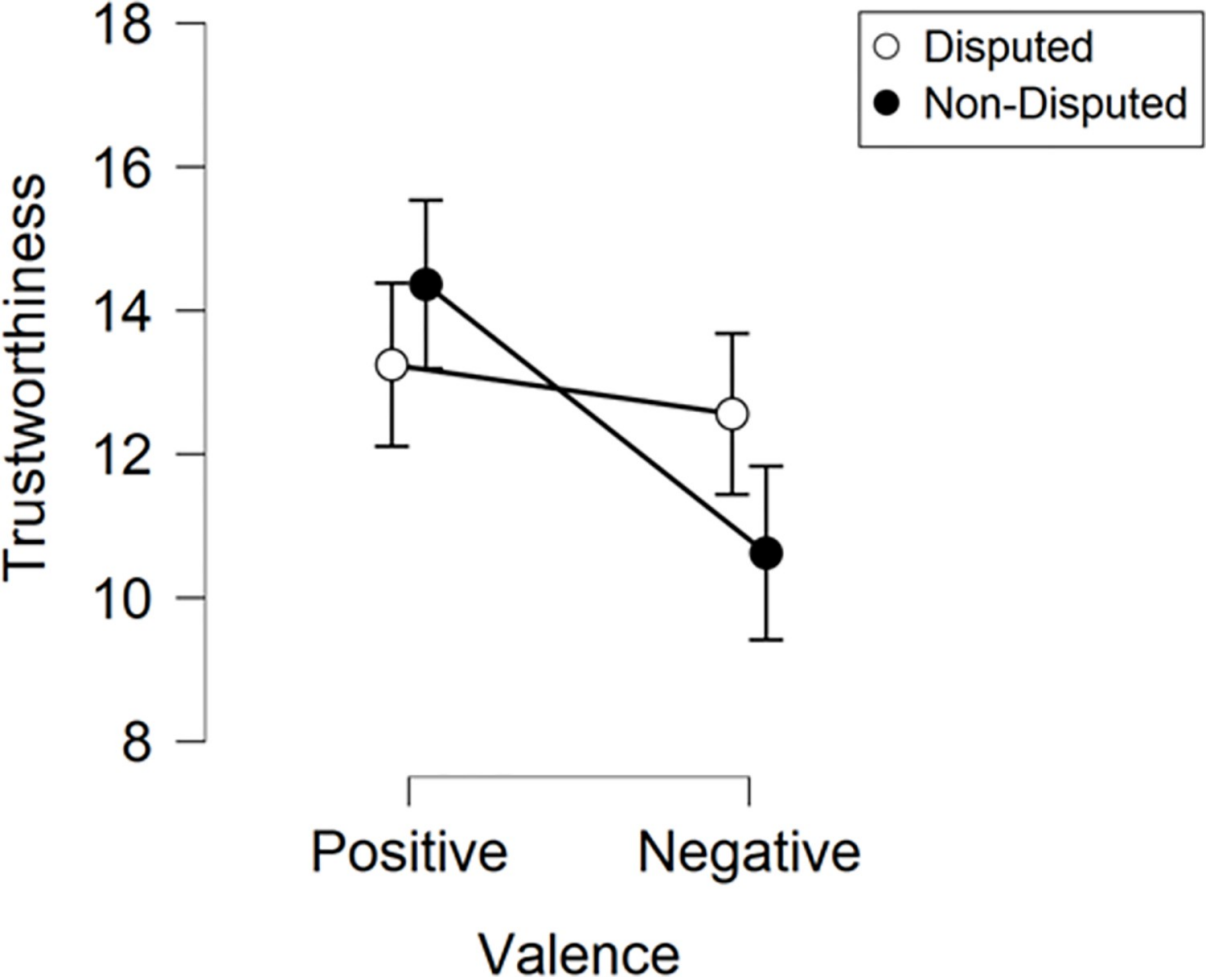
Experiment 1 ratings. Trustworthiness ratings obtained during the test phase of Experiment 1. Error bars depict ±SEM.

The trustworthiness ratings obtained during the test phase were subjected to ANOVA with Valance (positive, negative) and Credibility (disputed, non-disputed) as within-subjects factors (Fig 2). The analysis revealed a significant main effect of Valence [*F*(1,127) = 11.40, *p* < .001, η_p_^2^ = .08], whereby targets associated with positive tweets (*M* = 13.80) were rated as more trustworthy than targets associated with negative tweets (*M* = 11.59). There was no main effect of Credibility [*F*(1,127) = 0.98, *p* = .325, η_p_^2^ = .01]. However, there was a significant Valence × Credibility interaction [*F*(1,127) = 6.34, *p =* .013, η_p_^2^ = .05]. Follow up t-tests, with a Bonferroni-adjusted alpha level of .025 (.05/2), were conducted to explore this interaction. Results showed that trustworthiness ratings of faces paired with positive disputed (*M* = 13.25) and negative disputed headlines (*M* = 12.56) did not differ significantly [*t*(127) = 0.89, *p* = .378, d = 0.08]. However, the trustworthiness ratings of faces paired with positive non-disputed headlines (*M* = 14.36) and negative non-disputed headlines (*M* = 10.62) did differ significantly [*t*(127) = 3.75, *p* < .001, d = 0.33]. This suggests that the modulation of facial trustworthiness induced by headline valence is attenuated by the presence of disputed tag.

#### Recall of headline valence

Next, we sought to assess participants’ recall of the valence of the headline associated with each target face. Participants’ confidence ratings were subjected to ANOVA with Valence (positive, negative) and Credibility (disputed, non-disputed) as within-subjects factors. The analysis revealed a significant main effect of Valence [*F*(1,127) = 80.91, *p* < .001, η_p_^2^ = .39], whereby participants were more confident that targets were paired with positive headlines when they were paired with a positive headline (*M* = 2.09), than when paired with a negative headline (*M* = -8.33). This suggests participants had some recollection of headline valence. A significant effect of Credibility was also found [*F*(1,127) = 5.90, *p* = .017, η_p_^2^ = .04], with participants more confident that the headlines were positive if they were non-disputed (*M* = -2.01), than if they were disputed (*M* = -4.23). We observed no Valence × Credibility interaction [*F*(1,127) = 0.92, *p* = .340, η_p_^2^ = .01].

#### Recall of headline credibility

Finally, we sought to assess participants’ recall of the credibility of the headline associated with each target face. Participants’ confidence ratings were subjected to ANOVA with Valence (positive, negative) and Credibility (disputed, non-disputed) as within-subjects factors. The analysis revealed a significant main effect of Credibility [*F*(1,127) = 7.78, *p* = .006, η_p_^2^ = .06], with participants being more confident that targets were presented with a disputed headline when the headline was disputed (*M* = -8.42) than when the headline was not disputed (*M* = -11.01). This suggests that participants had some recollection of the headline credibility. We observed no main effect of Valence [*F*(1,127) = 2.41, *p* = .123, η_p_^2^ = .02], and no Valence × Credibility interaction [*F*(1,127) = 0.26, *p* = .612, η_p_^2^ = .002].

## Experiment 2

The results of Experiment 1 indicate that, in the absence of a disputed tag, participants tended to judge the faces associated with positive headlines as more trustworthy than those associated with negative headlines. When a disputed tag was present, however, target faces associated with positive and negative disputed headlines did not differ significantly in their perceived trustworthiness. These results suggest that the disputed tag may be effective in mitigating the potentially harmful effects of fake news on person evaluation.

These results accord with previous reports that people use disputed tags when deciding what to believe, and disregard information from less credible sources [39, 40]. They are, however, somewhat inconsistent with previous evidence that trait-relevant information influences person evaluation irrespective of source credibility [14, 48]. In our second experiment, we therefore sought to examine whether the ‘protective’ effects of the disputed tag are short-lived. It is conceivable, for example, that participants might find it difficult to hold in their memory which tweets were disputed and which were not. If their ability to ‘bind’ the disputed tag to the correct face-headline pairings was tenuous, increasing the interval between the study and test phases might render the disputed tags less effective.

### Method

#### Participants

Protocols were approved by the University of York’s Psychology Ethics Committee, participants gave informed written consent before taking part. One-hundred-and-twenty-eight participants completed the experiment (*M*_age_ = 29.12, *SD*_age_ = 9.36; 78 female, 44 male, 4 non-binary, 2 prefer not to say), recruited via Prolific. A further 42 were tested, but replaced having reported that they did not notice the disputed tag. Of the 128 participants in the final sample, all reported English as their first language and 126 resided in the UK (one resided in Portugal and one in Ireland). Of these 128 participants, 110 identified as White British, 1 as Black British, 2 as White and Black Caribbean, 1 as White and Black African, 4 as White and Asian, 3 as Indian, 2 as Pakistani, 1 as Chinese, 1 as “other” Asian background (not specified), 1 as African, and 1 as Caribbean. All participants received a small honorarium (£3.50) for their participation.

#### Design and procedure

The design of Experiment 2 was almost identical to that of Experiment 1. The only change was the inclusion of a 10-minute delay between the initial study phase and the test phase. Participants completed a distractor task during this interval, in which they were asked, in turn, to list as many uses as possible for five objects (a wellington boot, blanket, brick, watering can and a paperclip). Participants were given two minutes per object with the trustworthiness rating phase presented directly after.

### Results

The data for this experiment, and pre-registered hypotheses and analysis plans, are available open access at Open Science Framework (https://osf.io/t7c25/?view_only=8e6ca4f1249c4579a0e49e1ce5f4d747).

### Believability ratings

The believability ratings obtained during the study phase were subjected to a within subjects ANOVA with Valence (positive, negative), Credibility (disputed, non-disputed), and Presentation (first, second) as within-subjects factors. The analysis revealed a significant main effect of Credibility [*F*(1,127) = 107.53, *p* < .001, η_p_^2^ = .46], whereby non-disputed tweets (*M* = 15.46) were rated as more believable than disputed tweets (*M* = -6.90). There was also a significant main effect of Valence [*F*(1,127) = 69.82, *p* < .001, η_p_^2^ = .36], whereby positive tweets (*M* = 9.28) were rated as more believable than negative tweets (*M* = -0.72). A significant Credibility × Presentation interaction was observed [*F*(1,127) = 19.74, *p* < .001, η_p_^2^ = .13]. Simple contrasts revealed that disputed tweets at presentation one (*M* = -5.49) were judged as more believable than disputed tweets at presentation two (-8.30) (*p* < .001). In contrast, non-disputed tweets at presentation one (*M* = 14.54) were seen as less believable than non-disputed tweets at presentation two (*M* = 16.38) (*p* = .038). A significant Valence × Presentation interaction was also found [*F*(1,127) = 9.27, *p* = .003, η_p_^2^ = .07]. Follow up t-tests, with a Bonferroni-adjusted alpha level of .025 (.05/2), were conducted to explore this interaction. Results revealed that positive tweets were judged more believable at presentation one (*M* = 10.13) than at presentation two (*M* = 8.43) [*t*(127) = 2.83, *p* = .005, d = 0.25]. The believability ratings of negative tweets at presentation one (*M* = -1.08) and presentation two (*M* = -0.35) did not differ significantly [*t*(127) = -1.34, *p* = .182, d = -0.12]. There was no main effect of Presentation [*F*(1,127) = 1.40, *p =* .*239*, η_p_^2^ = .01], no Valence × Credibility interaction [*F*(1,127) = .21, *p* = .645, η_p_^2^ = .002], and no Valence × Credibility × Presentation interaction [*F*(1,127) = .12, *p* = .730, η_p_^2^ = < .001].

### Trustworthiness ratings

**Fig 3:**
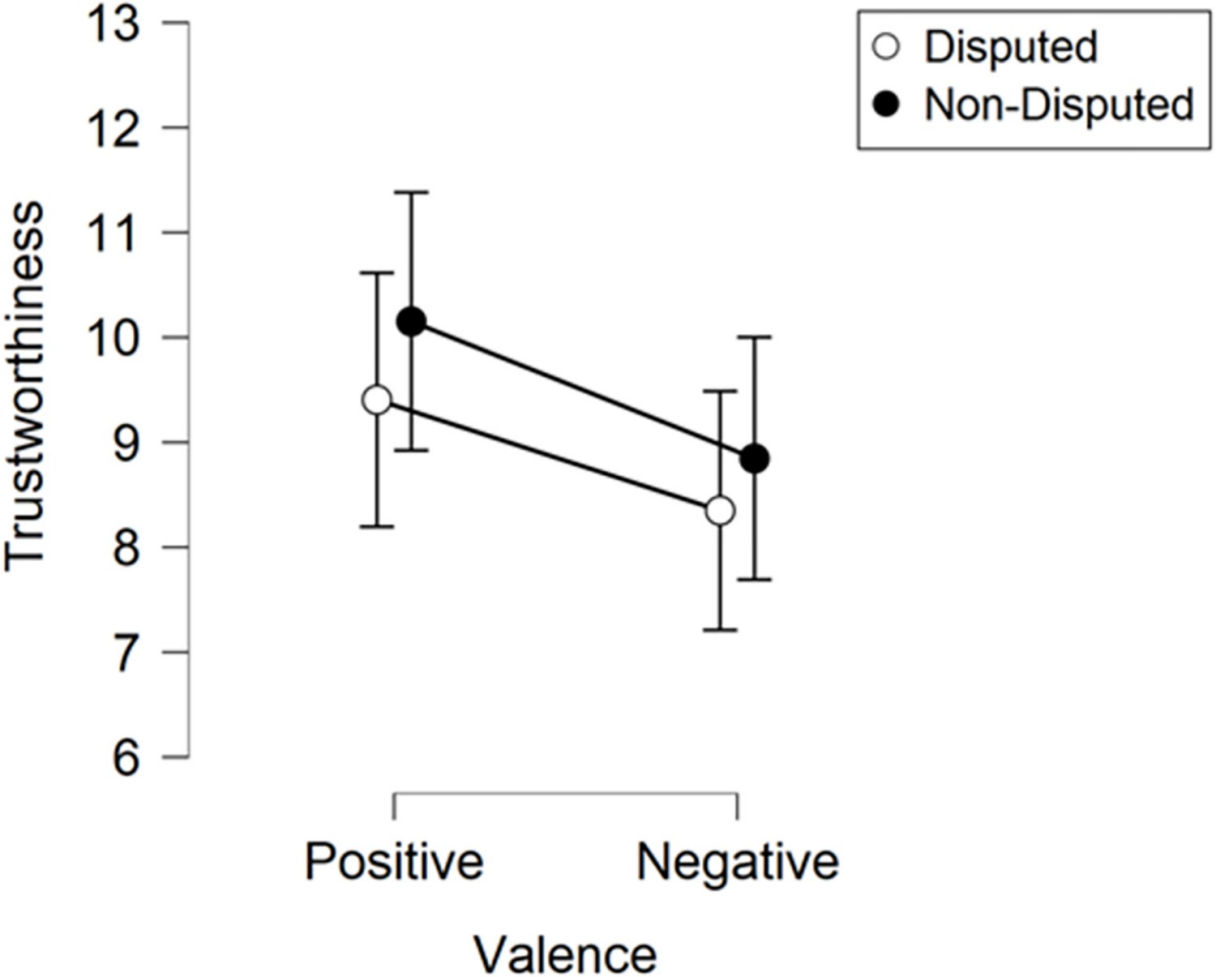
Experiment 2 ratings. Trustworthiness ratings obtained during the test phase of Experiment 2. Error bars depict ±SEM.

The trustworthiness ratings obtained during the test phase were subjected to ANOVA with Valence (positive, negative) and Credibility (disputed, non-disputed) as within-subjects factors (Fig 3). The analysis revealed a significant main effect of Valence [*F*(1,127) = 5.09, *p* = .026, η_p_^2^ = .04], whereby targets associated with positive headlines (*M* = 9.78) were rated as more trustworthy than targets associated with negative headlines (*M* = 8.60). We observed no main effect of Credibility [*F*(1,127) = 1.17, *p* = .281, η_p_^2^ = .009] and no Valence × Credibility interaction [*F*(1,127) = .04, *p* = .840, η_p_^2^ = < .001].

### Recall of headline valence

Next, we sought to assess participants’ recall of the valence of the headline associated with each target face. Participants’ confidence ratings were subjected to ANOVA with Valence (positive, negative) and Credibility (disputed, non-disputed) as within-subjects factors. The analysis revealed a significant main effect of Valence [*F*(1,127) = 85.83, *p* < .001, η_p_^2^ = .40], whereby participants were more confident that targets were paired with positive headlines when they were paired with positive headlines (*M* = 4.19) than when they were paired with negative headlines (*M* = -6.40). This suggests participants had some recollection of headline valence. A significant effect of Credibility was also found [*F*(1,127) = 11.36, *p* = .001, η_p_^2^ = .08], with participants more confident that headlines were positive when they were non-disputed (*M* = 0.34) than when they were disputed (*M* = -2.55). There was no Valence × Credibility interaction [*F*(1,127) = 1.76, *p* = .187, η_p_^2^ = .01].

### Recall of headline credibility

Finally, we sought to assess participants’ recall of the credibility of the headline associated with each target face. We subjected participants’ confidence ratings to ANOVA with Valence (positive, negative) and Credibility (disputed, non-disputed) as within-subjects factors. The analysis revealed a significant main effect of Credibility [*F*(1,127) = 7.57, *p* = .007, η_p_^2^ = .06], with participants being more confident that targets were presented with a disputed headline when the headline was disputed (*M* = -5.13) than when the headline was non-disputed (*M* = -7.47). We observed no main effect of Valence [*F*(1,127) = 3.50, *p* = .064, η_p_^2^ = .03] and no Valence × Credibility interaction [*F*(1,127) = 0.62, *p* = .432, η_p_^2^ = .005].

## General discussion

Adults spontaneously attribute a wide range of traits to others based on their facial appearance [1, 2]. Previous findings indicate that this person evaluation is affected by information provided about the targets’ previous actions and behaviours [12–15]. The present study sought to determine whether Twitter posts that pair facial images with favourable and unfavourable biographical information afford comparable face-trait learning. We also sought to establish whether any effects of the biographical information provided were attenuated by the presence of the “disputed tag” used by Twitter to mitigate the impact of fake news. To this end, participants were shown fictious tweets that paired strangers’ faces with news headlines suggestive of positive or negative behaviours. Half of the tweets were tagged as ‘disputed’ and half were untagged.

In our first experiment, we found that, in the absence of a disputed tag, participants tended to judge targets associated with positive headlines to be more trustworthy than those associated with negative headlines. Interestingly, when a disputed tag was present, participants judged targets associated with positive and negative headlines as similarly trustworthy. These results appear to suggest that disputed tags are effective in mitigating the potentially harmful effects of fake news on person perception. In our second experiment, however, we found that the ‘protective’ effects of the disputed tag disappeared when a 10-minute delay was introduced between the study phase and test phase. Under these conditions, we found that headline valence modulated subsequent trust ratings irrespective of whether they were accompanied by a disputed tag.

Broadly speaking, our results–in particular, those of the Experiment 2 –accord with previous findings described by Baum and colleagues which suggest that trait-relevant information influences person evaluation irrespective of source credibility [14, 47]. Although we observed an effect of the disputed tag in Experiment 1, the effect of the credibility manipulation appears to be so short-lived as to have little impact on face-trait learning seen outside the lab. If the presence of a disputed tag has little or no effect after 10 minutes, it is unlikely to have an effect after 10 hours or 10 days. Nevertheless, this pattern of results raises the question: why do the effects of headline-valence survive a 10-mins delay, while the effects of the headline-credibility do not?

Our findings, and those from previous studies [12, 13, 15, 47], suggest that faces readily acquire positive and negative valence when paired with information about the individuals’ positive and negative behaviours. This kind of learning may be relatively fast and easy, even to the point of being hard to inhibit. One possibility is that the natural relational structure between “agent” and “action” makes the elements easy to associate (e.g., they can be readily visualised). Another factor may be that the actions used here (e.g., “Local woman saves boy from drowning in river”) and elsewhere in the literature (e.g., “Stole money and jewellery from the relatives he was living with”[13]) are emotive and salient. Finally, this kind of learning may occur even where participants encode only the gist or valence of the past behaviour.

While face-action or face-valence learning may be fast and easy, face-credibility or information-credibility learning may be harder. The relational structure between the to-be-learned elements is perhaps less intuitive (e.g., harder to visualise). The disputed tags used by Twitter are also relatively subtle and may fail to capture participants’ attention. Indeed, in both experiments, a non-trivial number of participants had to be replaced for having failed to notice the presence of the disputed tags. As a result, we speculate that the learning responsible for the credibility effect in Experiment 1 was vulnerable to subsequent interference and soon started to decay [44].

The present findings have some disturbing implications for the effectiveness of propaganda circulated via social media. Our results suggest that fake news stories that depict an individual alongside negative biographical information (e.g., fictitious descriptions of their previous misdemeanours, crimes, and anti-social behaviour) may well have detrimental effects on the way that person is evaluated by users who encounter this content. The results from our second study suggest that the presence of a disputed tag may do little to reduce the impact on users’ perceptions of those depicted. Perceptions of trustworthiness are thought to exert a strong influence on our decision making [49], they may affect how we vote [4], and what kinds of punishments we endorse [11]. Given the reach of social media platforms like Twitter, the dangers posed by this kind of propaganda are obvious.

We should also be mindful of the dangers posed by more subtle propaganda campaigns. As discussed above, it is well-established that participants quickly learn that individuals are trustworthy or untrustworthy when their faces are paired with details about their supposed pro-social and anti-social behaviours. Importantly, however, this kind of face-trait learning is known to generalise to other individuals who resemble those encountered during the study phase [16, 17, 19, 50]. In other words, if we learn that Bob is untrustworthy, we may spontaneously dislike Bob’s cousin Fred, with whom he shares a passing resemblance. Together, these findings suggest a sinister possibility; that it may be possible to alter the public perception of a rival by circulating fake news stories on social media that present people who resemble the rival in unflattering terms. Conceivably, this kind of propaganda may pass unnoticed unless one suspected someone was being targeted in this way and was purposely searching for it.

It is also important that future research consider if and how the impact of disputed tags and other warning labels can be enhanced. Recent research suggests that placing warning labels immediately after, rather than alongside, a false headline may have a more powerful and longer lasting effect on its believability. According to the ‘concurrent storage hypothesis’, valence information and credibility are initially retained but message credibility fades more quickly from memory over time. Presenting the credibility label after the misinformation may help to increase its salience and so retention [44]. Other work has investigated the content of the warnings themselves, showing that the nature and appearance of the warning labels may influence their effectiveness [40] For example, Kirchner & Reuter [41] showed that adding an explanation for why a post had been labelled as disinformation increased its influence on perceived accuracy [41]. It would be interesting to measure whether manipulating the content of warning labels to enhance their salience increases their protective effects on person perception. If warning labels cannot be made more effective, there may be a case for blocking or obscuring the facial images included in disputed stories.

It is important to acknowledge that our highly controlled experimental design differs in important respects from more naturalistic viewing conditions. For example, in our design participants viewed each face separately for a pre-specified time. It would be interesting to investigate face-trait learning in conditions more closely resembling those of twitter where participants can scroll through multiple stories at their leisure. In our design, we also blurred out information regarding the people who sent the tweets. In future research, it would be interesting to measure how the legitimacy of the source interacts with misinformation tags [51]. It would also be interesting to investigate how engagement with the disputed tag is influenced by the group membership and pre-existing biases of the participants. Finally, previous work has demonstrated that ambiguous behaviours are more likely to be interpreted as negative when the individual depicted has a face that is perceived as untrustworthy [52]. With this in mind, it may be interesting to investigate how the valence of the tweet, the presence of the disputed tag, and the perceived trustworthiness of an individual depicted may interact. It may be that participants would give more credence to the disputed tag when the information was negative and the individual depicted appeared untrustworthy than when the information was negative but the individual depicted appeared trustworthy. Addressing these questions will help us understand how best to combat misinformation online.

Across two experiments, we found that fictitious tweets that paired facial images with details of the person’s positive or negative actions affected the extent to which readers subsequently judged the faces depicted to be trustworthy. When the rating phase followed immediately after the study phase, the presence of disputed tag attenuated the effect of the behavioural information. However, when the rating phase was conducted after a 10-mins delay, the presence of disputed tag had no effect. Our findings suggest that disputed tags may have relatively little impact on face-trait learning that occurs via social media. As such, fake news stories may have considerable potential to shape users’ person evaluation.
